# Imaging Findings and Toxicological Mechanisms of Nervous System Injury Caused by Diquat

**DOI:** 10.1007/s12035-024-04172-x

**Published:** 2024-04-15

**Authors:** Yanguang Ren, Feng Guo, Lin Wang

**Affiliations:** Department of Emergency Medicine, Shengjing Hospital of China Medical University, Tiexi District, No. 39 Huaxiang Road, Shenyang, 110000 Liaoning People’s Republic of China

**Keywords:** Diquat, Poisoning, Nervous system, Mechanisms of intoxication, Oxidative stress

## Abstract

Diquat (DQ) is a nonselective bipyridine herbicide with a structure resembling paraquat (PQ). In recent years, the utilization of DQ as a substitute for PQ has grown, leading to an increase in DQ poisoning cases. While the toxicity mechanism of DQ remains unclear, it is primarily attributed to the intracellular generation of reactive oxygen species (ROS) and reactive nitrogen species (RNS) through the process of reduction oxidation. This results in oxidative stress, leading to a cascade of clinical symptoms. Notably, recent reports on DQ poisoning have highlighted a concerning trend: an upsurge in cases involving neurological damage caused by DQ poisoning. These patients often present with severe illness and a high mortality rate, with no effective treatment available thus far. Imaging findings from these cases have shown that neurological damage tends to concentrate on the brainstem. However, the specific mechanisms behind this poisoning remain unclear, and no specific antidote exists. This review summarizes the research progress on DQ poisoning and explores potential mechanisms. By shedding light on the nerve damage associated with DQ poisoning, we hope to raise awareness, propose new avenues for investigating the mechanisms of DQ poisoning, and lay the groundwork for the development of treatment strategies for DQ poisoning. Trial registration number: 2024PS174K.

## Introduction

Diquat (DQ) belongs to the class of nonselective bipyridine herbicides, sharing a structural similarity with paraquat (PQ). It ranks as the third-largest herbicide globally, following PQ and glyphosate usage. In 1955, DQ was initially synthesized by the British company ICI, which later recognized its value as a desiccant and herbicide, leading to its market introduction in 1958 [1]. While DQ’s usage is not as widespread as PQ, the absence of specific antidotes for PQ poisoning and its high mortality rate have prompted many countries worldwide to ban the sale and use of PQ. Consequently, DQ has seen increased utilization as a substitute for PQ in recent years, accompanied by a rise in DQ poisoning cases. A study conducted from 2004 to 2007, focusing on accidental pesticide exposure in children in the USA, identified DQ as one of the most common accidental pesticide exposures among children [2].

DQ poisoning can inflict damage on multiple tissues and organs, with the digestive tract and kidneys being the most frequently affected, followed by the liver, lungs, and heart. It is crucial to note that DQ also exerts toxic effects on the central nervous system, leading to clinical symptoms such as dizziness, drowsiness, convulsions, coma, irritability, and disorientation [3]. When a patient presents with neurological symptoms, DQ often predicts the disease’s severity and a poor prognosis. Currently, most research on DQ poisoning centers on the primary target organs like the kidneys, liver, and digestive tract, with relatively fewer studies on central nervous system injury. The mechanism underlying nerve injury remains unclear, and effective treatments for DQ-induced neurological injury are lacking. This review consolidates research progress on DQ, encompassing its physicochemical properties, poisoning methods, neurological damage, imaging manifestations, and potential mechanisms. Our aim is to propose fresh insights into the mechanism of DQ poisoning and provide a theoretical foundation for developing treatment strategies for nervous system injuries arising from DQ poisoning.

## Physicochemical Properties of DQ

DQ is an odorless yellow crystal, also known as 1,1′-ethylene-2,2-bipyridylium dibromide. It has a relative molecular weight of 344.05 and is insoluble in nonpolar organic solvents. It is slightly soluble in methanol and easily soluble in water at room temperature. Typically, it is prepared as a dark brown transparent liquid with an earthy odor [3]. DQ is relatively stable in neutral and acidic solutions but becomes unstable when exposed to light in alkaline solutions. It is prone to hydrolysis, with the rate of hydrolysis increasing with higher temperatures. You can find specific physical and chemical properties of DQ in Table 1. DQ is known for its effective weed control properties. When absorbed by green plant tissues, it generates peroxide free radicals, leading to damage and rapid cell death in plant membranes. As a result, DQ is widely used for weed control purposes.

**Table 1:**
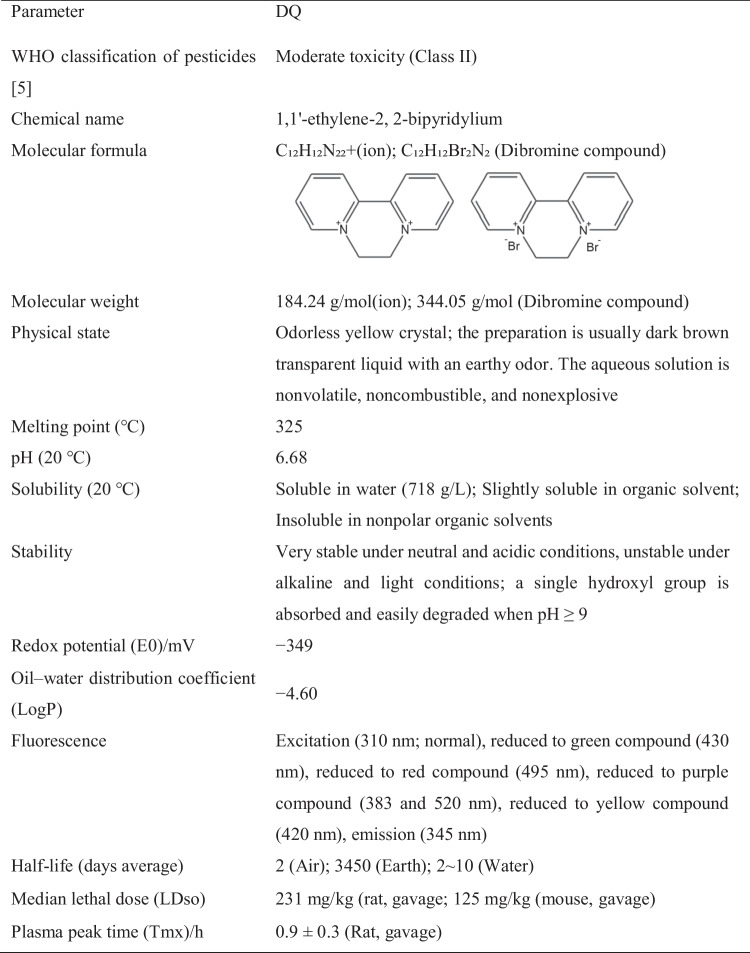
Physicochemical properties of DQ (References [3, 75])

## Poisoning Method of DQ

DQ can be absorbed by the gastrointestinal, respiratory, eye, or mucocutaneous routes. It has also been reported in the literature to be absorbed by subcutaneous, intramuscular, and vaginal injections, although these occurrences are rare [4, 5]. DQ is less absorbed through the skin and lungs. The skin effectively prevents DQ absorption, but DQ absorption increases when the skin is damaged [6]. Gastrointestinal ingestion is the most common and most damaging route of acute DQ poisoning. In the gastrointestinal tract, DQ is degraded by intestinal flora, resulting in an absorption rate of less than 10% [7]. After being absorbed into the human body, DQ is rapidly distributed to various organs and tissues through the bloodstream [8]. Among these, the concentrations in the liver and kidney are high, with distribution in the liver, kidney, gastrointestinal tract, lung, and other tissues peaking at 2 h and then decreasing rapidly [3]. While the absorption rate of DQ is low, it is rapidly and widely distributed. Autopsies of DQ poisoning patients have shown varying concentrations of DQ in tissues (body fluids) of patients who died at different time intervals. For instance, for patients who died at 14 h, the order of concentration from high to low was urine > vitreous humor in the eye > lungs > liver > brain tissue > kidneys [9]. In contrast, for patients who died at 143 h, the concentrations in descending order were liver > skeletal muscle > lung > kidney > brain tissue > heart [10].

DQ remains relatively stable in the human body. A small amount undergoes pyridine epoxidation in the liver through cytochrome P450 enzymes, producing less toxic iodopyridine and dipyridine derivatives, which are further metabolized [11]. Most of the DQ entering the digestive tract is excreted in feces within 24 h (approximately 90–95%), with around 45% excreted in urine within 48 h of absorption. A negligible amount is excreted in bile [3, 11], and only approximately 2% of DQ is absorbed into the blood and distributed to various tissues. Some reports suggest no significant difference in the reduction of urinary PQ and DQ poisoning when comparing the two. Still, the DQ concentration in bile tends to be higher, indicating that bile may reduce DQ concentrations in serum and human tissues after long-term ingestion [12]. DQ can have a positive effect on the reductive-oxidative activity of organisms when ingested rapidly, and in severe cases, it can lead to sudden death.

## Neurologic Injury and Imaging Findings of DQ Poisoning

Acute DQ toxicity often results in multiorgan insufficiency. Unlike PQ, DQ causes mild reversible damage to type I lung cells and does not damage type II alveolar cells [13]. Animal experiments have confirmed that the half-life of DQ in the lungs is one-sixth that of PQ [14], making the lungs less susceptible to damage. However, it is important to note that DQ has a toxic effect on central nervous system cells, leading to relatively common and severe central nervous system symptoms [15], which can manifest as recurrent headaches, dizziness, drowsiness, coma, epileptiform seizures, and more, often with a high mortality rate. Animal experiments have shown that oxidative stress in the brain leads to degeneration of dopaminergic cells [16], resulting in over an 80% reduction in dopamine uptake [17]. In an experiment by Wu et al. [18], Wister rats infected with DQ by gavage exhibited symptoms such as listlessness, disheveled fur, reduced mobility, and altered breathing patterns in the early stages of poisoning. Some mice even showed signs of central nervous system damage with uncoordinated limb movements before and after poisoning.

In clinical cases published as early as 1981, Vanholder et al. [15] reported the case of a 64-year-old patient with DQ poisoning who suffered intracerebral hemorrhage and later died in a coma. Autopsy results revealed pontine hemorrhage and brainstem infarction as the causes of death. In 1999, Rudez et al. [4] reported a patient who experienced quadriplegia and dysarthria 3 months after a vaginal injection of 20 mL of DQ. Saeed et al. [19] documented a case of DQ poisoning leading to intracranial hematoma in the right basal ganglia region in 2001. In 2020, Xing et al. [20] reported that a 21-year-old male patient with DQ poisoning developed impaired consciousness and bilateral Babinski signs after 5 days of symptomatic treatment. Head CT scans on day 11 showed acute pontine demyelination. Notably, there were abnormal signals in the pons, as observed in diffusion-weighted and apparent diffusion coefficient imaging. The patient’s serum sodium concentration was normal, ruling out sodium-related demyelination. This study emphasized the diagnostic value of neuroimaging for central pontine myelinolysis. Cochinwala et al. [21] reported a case in a 2-year-old boy in 2021, where a previously healthy Hispanic boy mistakenly ingested DQ. On the third hospital day, a CT scan of the brain showed reduced density in the brainstem and central gray matter, diffuse intracerebral edema, interval increase in the size of the lateral and third ventricles, and effacement of the suprasellar and basilar cisterns, indicating uncal, hippocampal, and tonsillar herniation. Wang et al. [22] measured the concentration of DQ in the cerebrospinal fluid of patients with DQ poisoning and found that the concentration of DQ in the cerebrospinal fluid decreased significantly faster than in the blood. Importantly, a specific correlation was observed between the concentration of DQ in the cerebrospinal fluid, intracranial pressure, and the severity of cerebral edema in this patient.

**Fig. 1:**
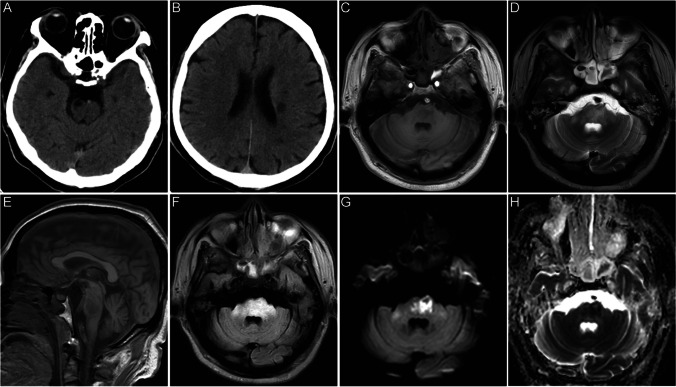
Neuroimaging findings in DQ-induced toxic encephalopathy. A head CT scan showed patchy low-density shadows on the right side of the brainstem (**A**) and near the left ventricle (**B**). Multiple patchy long T1 (**C**) and long T2 (**D**) signals in the right frontal lobe, bilateral paraventricular, bilateral basal ganglia, brainstem, and right cerebellum. Large low-signal shadows in the T1W sagittal position (**F**) pons. FLAIR mostly showed high signal (**F**). DWI (**G**) brainstem lesions showed significantly high signal, and ADC (**H**) showed low signal

Chen [23] reported that imaging of the DQ-poisoned brain revealed symmetrical lesions in the cerebellar dentate nucleus, pontine brain, and deep nuclei in the basal ganglia region, with the pontine and basal ganglia regions being more affected. Particularly, the lesions in the pontine encepontica were characterized by symmetrical lesions in the center of the pons that did not involve the surrounding pons tissue and corticospinal tract. This finding suggests that the pons is a characteristic lesion site in DQ toxic encephalopathy and holds important differential diagnostic significance. In recent years, with the widespread use of DQ in China, Chinese scholars have reported several cases of neurological damage associated with DQ poisoning, as shown in Table 2 [20, 22–25]. In September 2023, the emergency department of Shengjing Hospital, affiliated with China Medical University, where the author works, also admitted a patient with nervous system damage after DQ poisoning. This 51-year-old woman ingested approximately 120 mL of DQ (20 g/100 mL), which was followed by nausea and vomiting. Her heart rate was 107 beats/min on admission, and her blood pressure was 178/105 mmHg. Laboratory test results were as follows: white blood cell (WBC), 12.03 × 10^9^/L; alanine aminotransferase (ALT), 10 IU/L; aspartate aminotransferase (AST), 12 IU/L; creatinine, 47.6 mol/L; urea, 7.6 mmol/L; potassium, 3.75 mmol/L; sodium, 134 mmol/L; and blood DQ concentration, 3.59 ng/mL. A head computed tomography scan at the time of admission showed patchy hypodense opacities on the left side of the ventricle and on the right side of the brainstem, which were considered to be old lesions in the patient’s brain. On the third day after admission, the patient became unconscious. On the 15th day after admission, head MRI scans showed multiple patchy long T1 and long T2 signal shadows in the right frontal lobe, bilateral paraventricles, bilateral basal ganglia, brainstem, and right cerebellum, with most of the FLAIR images appearing hyperintense. DWI showed that the brainstem lesions were significantly hyperintense, with corresponding ADC showing hypointensity (Fig. 1). After 43 days of hospitalization, the patient was discharged with the ability to lift her left limb off the bed but not against resistance.

**Table 2:** Cases of DQ poisoning

Case	Sex	Age	DQ concentration	Dose (mL)	Blood DQ concentration(ng/mL)	CT findings	MRI findings	Prognosis	References
1	F	16	20%	120	63.7	Decreased diffuse density in the brain stem	Pons showed long T1 and short T2 signals, DWI and FLAIR showed low signals	Improvement in clinical symptoms	26
2	M	20	20%	80–100	0.93	——	MRI showing T1WI, T2WI, T2-FLAIR, and DWI of the brainstem, particularly in the pons, following central pontine myelinolysis signal intensity	After 3 months, he continued to experience dystaxia and difficulty in walking	27
3	M	20	20%	80–100	——	A low density in the brainstem region	MRI showed abnormal high intensity in the pons, bilateral brachium pons, and pedunculus cerebri on T1WI, T2WI, and T2-FLAIR, and slight high intensity on DWI	After 18 days, he experienced cardiac arrest and died	27
4	M	31	20%	50	0.43	——	T1WI, T2WI, T2-FLAIR, and DWI depict abnormal medulla oblongata, pons, midbrain, cerebellum, bilateral brachium pons, and pedunculus cerebri	At 57 days, his symptoms were almost completely relieved	27
5	M	21	20%	100	——	——	Pontine lesion, which appeared hyperintense on T2-weighted imaging and fluid-attenuated inversion-recovery imaging	At 15 days after admission, the patient died of multiple organ dysfunction syndrome	22
6	F	55	20%	50	——	CT was normal on admission	Pons on T1WI was observed with patchy low-signal shadow in pons. Pons on T2WI showed a flaky and slightly high signal shadow. The T2-FLAIR lesions are significantly hyperactive	Clinical symptoms and imaging findings improved	25
7	F	32	20%	100	——	Decreased pons density	DWI showed a marked pattern of high signal shadow in the pons with limited diffusion	Clinical symptoms and imaging findings improved	25
8	M	51	20%	120	3.59	Left ventricle and right brainstem softened lesion	Patchy long T1 and long T2 signal shadows were observed in the right frontal lobe, bilateral lateral ventricle, bilateral basal ganglia, brainstem, and right cerebellum. FLAIR showed mostly high signals. The brain stem lesions on DWI showed high signal, and the ADC showed low-signal shadow	After 43 days of hospitalization, the patient was discharged with the left limb able to lift off the bed surface but not against resistance	
9	F	54	——	——	187.25	A hypodensity in the pons	——	After 26 days of therapy, the patient was discharged stable	24

## Mechanisms of DQ Nervous System Damage

In the past, most studies on DQ poisoning primarily focused on damage to organs such as the kidneys, liver, and lungs. However, in recent years, there has been a growing number of reports highlighting neurological damage resulting from DQ poisoning. This emerging issue has garnered increased attention within the scientific community. Although the precise mechanisms of DQ toxicity remain incompletely understood, its impact on the nervous system is multifaceted. Existing research indicates that DQ can generate numerous redox products through redox processes, exerting a potent toxic effect on central nervous cells and as a crucial factor contributing to nervous system damage.

### *Oxidative Stress Induced by DQ*

DQ, a nonselective bipyridine herbicide, exerts its detrimental effects on various tissues and organs primarily through the generation of copious amounts of ROS and RNS via redox reactions within cells, resulting in oxidative stress [26]. This oxidative stress subsequently leads to cellular dysfunction [27]. Due to DQ’s high redox potential, it possesses an enhanced capacity to induce oxidative damage [28].

Upon entering the body, DQ undergoes reduction facilitated by nicotinamide adenine dinucleotide phosphate (NADPH) and cytochrome P450 reductase (CPR). This reduction process, accompanied by electron transfer from NADPH, gives rise to NADP + and an exceedingly unstable DQ radical (DQ + ·). DQ + ·, in turn, donates electrons to molecular oxygen (O_2_), resulting in the formation of the superoxide radical (O_2_· −) and the restoration of DQ + · to its original state. The significant production of O_2_· − within this cycle prompts the generation of ROS, such as hydrogen peroxide (H_2_O_2_), either spontaneously or through superoxide dismutase (SOD) catalysis. Normally, H_2_O_2_ is converted into water via catalase (CAT) and glutathione peroxidase (GPX). However, when ROS levels exceed the body’s regulatory capacity, cellular protective mechanisms, encompassing nonenzymatic elements like glutathione (GSH), thioredoxin, selenium, and vitamins C and E, as well as antioxidant enzymes including SOD, GPX, GR, and CAT, become overwhelmed, resulting in oxidative stress [3, 17, 29, 30].

**Fig. 2:**
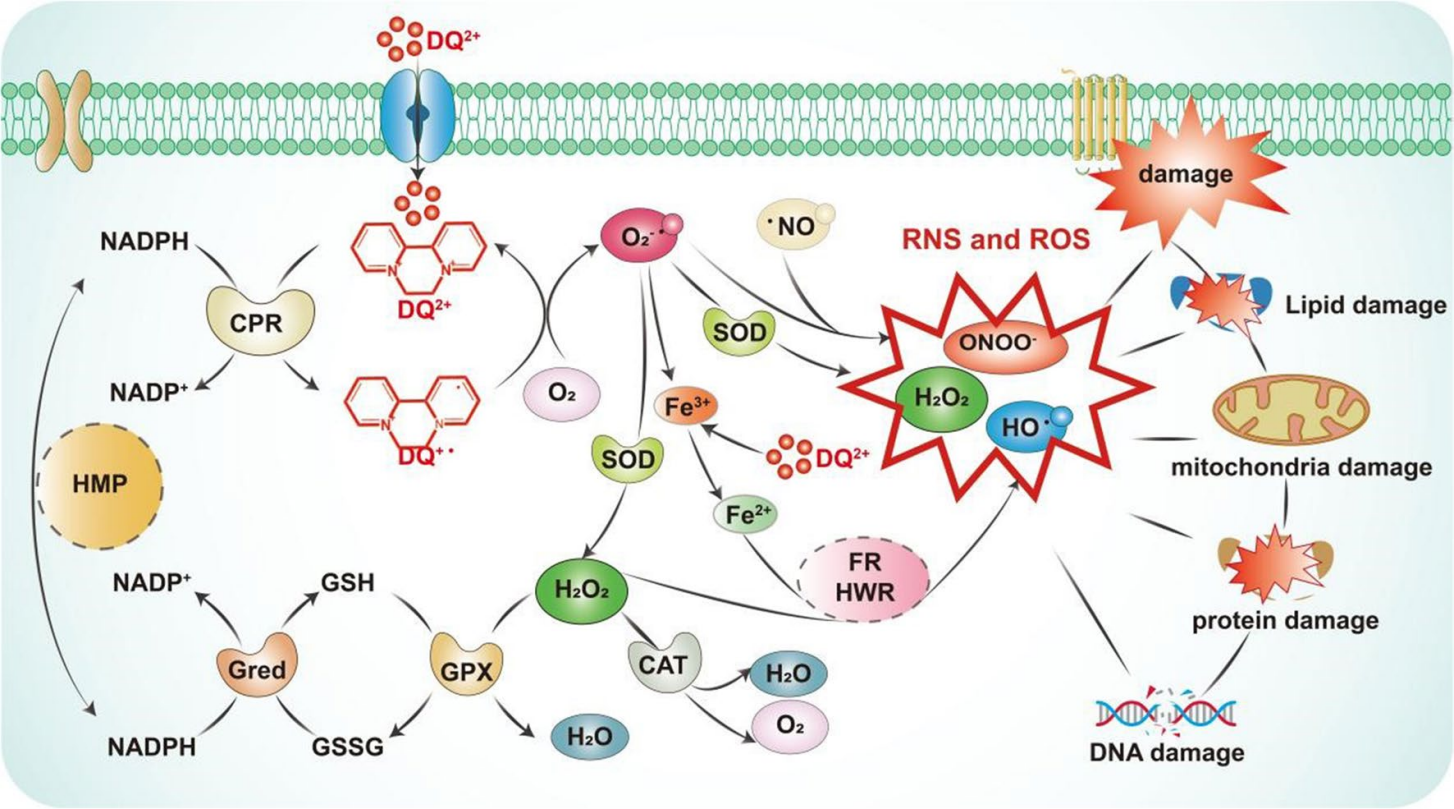
Role of DQ in inducing oxidative and nitrosative stress. DQ, diquat; ROS, reactive oxygen species; RNS, reactive nitrogen species; CRP, cytochrome P450 reductase; SOD, superoxide dismutase; NADPH, nicotinamide adenine dinucleotide phosphate; HMP, hexose monophosphate pathway; CAT, catalase; GPX, glutathione peroxidase; GSH, glutathione; GSSG, glutathione (oxidized); Gred, glutathione reductase; FR, Fenton reaction; HWR, Haber–Weiss reaction

Notably, DQ + · and O_2_· − can liberate iron from ferritin by reducing iron ions (Fe3 +) into ferrous ions (Fe2 +). These Fe2 + ions serve as catalysts in the Fenton reaction and the Haber–Weiss reaction, facilitating the conversion of H_2_O_2_ into the more potent hydroxyl radical (OH·), which wreaks havoc on biomolecules [31] (see Fig. 2). The accumulation of ROS within cells leads to DNA damage, while an abundance of hydroxyl radicals targets cellular lipid membranes, protein structures, and other macromolecules, resulting in structural damage to cell membranes and overall cellular harm. Additionally, the substantial consumption of CPR and NADPH leads to impairment of the cellular respiratory chain [32].

Moreover, DQ induces hypoxia within the body, leading to the heightened production of nitric oxide (NO). Under the influence of nitric oxide synthase (NOS), ROS and NO interact to generate peroxynitrite (ONOO^−^), which falls under the category of RNS. Excessive RNS levels can induce nitrosative stress, causing oxidative damage to intracellular lipids, proteins, and DNA [33]. ROS and RNS constitute vital small-molecule mediators within the body, and their excessive production disrupts the body’s antioxidant defense systems, triggering a severe oxidative stress response [34] (see Fig. 2).

### Autophagy: Maintaining Cellular Homeostasis

Autophagy is a highly regulated and evolutionarily conserved lysosome-mediated process responsible for protein degradation and organelle recycling [35]. In the central nervous system, autophagy plays a pivotal role in preserving the functional integrity of nerve tissue by acting as a cellular guardian against damage and ensuring cellular homeostasis by removing damaged or redundant organelles.

Within the autophagy pathway, the interplay of PTEN-induced putative kinase 1 (PINK1) and Parkin proteins is crucial in mitochondrial autophagy regulation. Studies have revealed that exposure of PC12 nerve cells to a DQ solution results in the inhibition of mitochondrial complex I activity by DQ, leading to changes in mitochondrial membrane potential [36]. This perturbation inhibits the entry of PINK1 into mitochondria, thereby triggering the accumulation of PINK1, which subsequently activates and recruits the Parkin protein [37]. Activated Parkin proteins facilitate the ubiquitination and degradation of mitochondrial outer membrane proteins [38, 39], ultimately culminating in the autophagic clearance of damaged mitochondria.

**Fig. 3:**
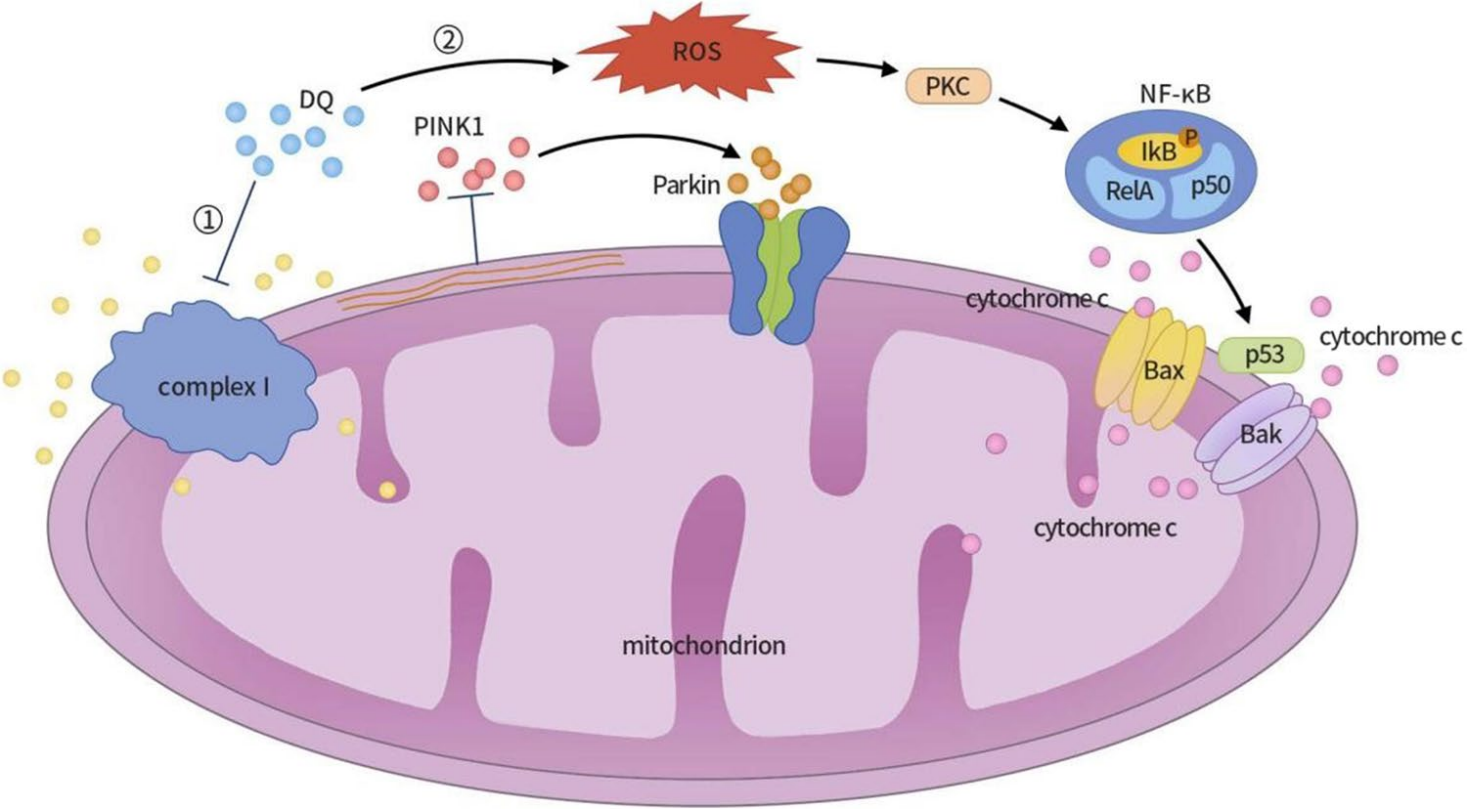
Mechanisms of DQ-induced neurotoxicity and apoptosis in PC12 nerve cells. (1) When PC12 nerve cells are exposed to a DQ solution, DQ inhibits mitochondrial complex I activity, causing changes in mitochondrial membrane potential and inhibiting PINK1 introduction to mitochondria. The accumulation of PINK1 triggers the activation and recruitment of Parkin protein, promoting the ubiquitination and degradation of mitochondrial outer membrane proteins, ultimately leading to the autophagic clearance of damaged mitochondria. (2) Reactive oxygen species (ROS) activate protein kinase C (PKC), which, in turn, phosphorylates protein IκB, leading to the dissociation of IκB from NF-κB and the subsequent activation of NF-κB. NF-κB regulates the expression of the pro-apoptotic factor P53, enhancing its expression in PC12 nerve cells and causing its accumulation in the cytoplasm and nucleus. When stress-induced damage becomes irreversible, P53 translocates to the mitochondrial outer membrane, triggering the release of mitochondrial pro-apoptotic genes Bax and Bak. Bax and Bak can form mitochondrial permeability transition pores, releasing cytochrome C, exacerbating mitochondrial dysfunction, and ultimately resulting in the apoptosis of PC12 nerve cells. DQ, diquat; PINK1, PTEN-induced putative kinase 1; ROS, reactive oxygen species; PKC, protein kinase C; NF-κB, nuclear factor-kappa B

Furthermore, DQ’s excessive ROS can activate protein kinase C (PKC). Activated PKC phosphorylates protein IκB [40], leading to the dissociation of IκB from nuclear factor-kappa B (NF-κB), thereby activating NF-κB. NF-κB orchestrates the regulation of the pro-apoptotic factor P53, ultimately augmenting P53 expression in PC12 nerve cells and causing its accumulation within the cytoplasm and nucleus [41]. When stress-induced damage becomes irreparable, P53 translocates to the mitochondrial outer membrane, releasing mitochondrial pro-apoptotic genes Bax and Bak. Bax and Bak each contribute to the formation of mitochondrial permeability transition pores (mPTP), culminating in the release of cytochrome C and the exacerbation of mitochondrial dysfunction. Ultimately, this cascade leads to apoptosis in PC12 nerve cells [42] (see Fig. 3).

Additionally, Cartelli et al. observed that the absence of Parkin protein in mouse cells resulted in microtubule over acetylation, inhibiting mitochondrial migration and transport [43]. In mice with Parkin deficiency, alterations in mitochondrial morphology and mitochondrial DNA (mtDNA) levels were evident, ultimately leading to degeneration and motor dysfunction of dopamine (DA) neurons [44, 45]. Parkin protein exists in a physiologically inhibited state, with activation occurring upon phosphorylation at the Serine65 (S65) site on its N-terminal ubiquitin-like (Ubl) domain [46]. Activated Parkin protein further facilitates autophagic clearance of damaged mitochondria by promoting ubiquitination and degradation of mitochondrial outer membrane proteins [47].

In summary, cellular damage inflicted by DQ may disrupt the delicate equilibrium within the autophagy process, representing a critical balance between cellular protection and the cytotoxic effects of DQ-induced stress and cellular damage. Park et al. reported that DQ activates NF-κB and p53 pathways to contribute to autophagy induction. Moreover, MAPK inhibitors control DQ-induced apoptosis and autophagy through mTOR signaling [48]. Fiesel et al. demonstrated the presence of mitophagy in the brains of Parkinson’s disease (PD) patients [49]. Kin et al. reported that DQ, when cytotoxic, induces both chaperone-mediated autophagy (CMA) and macroautophagy in SH-SY5Y cells. The induction of autophagy modulates cytotoxicity by suppressing apoptosis in DQ-exposed cells. CMA is vital in the degradation of pathological α-synuclein and the reduction of apoptosis in a DQ-induced α-synucleinopathy model [50].

### Nerve Cell Degeneration

Parkinson’s disease is a neurodegenerative condition characterized by a range of symptoms stemming from a reduction in the production of dopamine transmitters in the substantia nigra region of the midbrain, disrupting the equilibrium among neurons responsible for controlling movement. Key pathological features include Lewy bodies formation and the degeneration of dopamine-producing neurons [51]. In addition to genetic factors, there is mounting evidence suggesting that environmental factors also contribute to the onset and progression of Parkinson’s disease (PD). Epidemiological and toxicological investigations have pinpointed DQ as a major neurotoxic substance linked to the pathogenesis of PD [52–54]. In cases of acute DQ toxicity, it can exert severe toxic effects on the central nervous system, leading to symptoms resembling PD [55].

Numerous studies have demonstrated that DQ induces mitochondrial dysfunction and triggers an inflammatory cascade involving microglial activation by generating oxidative stress [16, 17, 56, 57]. These processes may be implicated in the degeneration of dopaminergic neurons within the substantia nigra. Lindquist et al. [58] injected 14C-labeled DQ into the abdominal cavity of temporal forest frogs and observed that performing whole-body autoradiography in mice revealed low and relatively uniform levels of radioactivity in brain tissue. Accumulation of DQ in neuromelanin could potentially result in lesions of pigmented nerve cells, contributing to the development of Parkinson’s disease.

In vitro studies conducted by Dafna et al. on primary midbrain cultures from Sprague–Dawley rats revealed that DQ-induced alterations in the morphology and quantity of dopaminergic neurons and reduced dopamine uptake [16]. Additionally, Singh et al. [54] explored the protective mechanism of standardized extracts of Bacopa monnieri against neurotoxicity induced by PQ and DQ. Their findings suggest that Bacopa monnieri may safeguard rat adrenal medulla pheochromoma differentiated cell line PC12 cells by modulating disrupted cellular redox pathways associated with PD, potentially offering therapeutic benefits in preventing Parkinson’s disease.

### Neuronal Axonal Degeneration

Neurons play a crucial role in the nervous system, transmitting information to other neurons or tissues via synapses. Among the components of neurons, the axon, also referred to as a nerve fiber, is the elongated projection responsible for conveying information from the cell body to other neurons or cells. Axonal degeneration is a process associated with neurodegenerative diseases like motor neuron disease [59], Alzheimer’s disease [60], and Huntington’s disease [61].

Fischer and Glass [62] conducted research confirming that oxidative stress constitutes a significant mechanism leading to axonal degeneration. They cultured dorsal root ganglion (DRG) neurons with a knockout of the SOD1 gene and observed significant axonal degeneration within 48 to 72 h. Importantly, the addition of SOD1 at this stage prevented such degeneration, thus validating that antioxidant treatment can mitigate this degeneration. Their study also assessed the sensitivity of wild-type DRGs to increased superoxide levels induced by DQ. The results demonstrated a positive correlation between axonal degeneration and the dose of DQ, with SOD1 deficiency exacerbating DQ’s toxicity. This finding suggests that DRG axons are susceptible to damage mediated by oxidative stress. DQ elevates intracellular superoxide production through oxidative stress, leading to axonal degeneration and degeneration. These results are a foundation for further investigations into oxidative stress-mediated axonal degeneration.

### Pontine Myelinolysis

Central pontine myelinolysis (CPM) is a demyelinating disorder most commonly associated with rapid correction of hyponatremia, although it can also be linked to alcoholism, malnutrition, and chronic liver disease [63–65]. In 2020, Xing et al. [20] reported a case of acute pontine demyelination in acute DQ poisoning, ruling out sodium-related demyelination. Currently, it is believed that CPM’s pathogenesis is tied to the swift correction of hyponatremia, resulting in an imbalance of water and electrolytes inside and outside brain cells, damage to the blood–brain barrier, and apoptosis and myelinolysis of glial cells within the central nervous system. However, the specific mechanism by which DQ leads to CPM remains unclear and may be related to oxidative stress following DQ poisoning, affecting the apoptosis of central nervous system glial cells. This hypothesis warrants further investigation.

## Treatment of DQ Nervous System Damage

Currently, there exists no specific drug for the treatment of DQ poisoning, emphasizing the importance of promptly implementing effective measures to remove the poison and enhance the excretion of absorbed toxins in cases of acute DQ poisoning. Hemoperfusion and hemodialysis represent effective methods for eliminating DQ from circulation. While the exact mechanism of neurological damage caused by DQ poisoning remains incompletely understood, it is widely believed that oxidative stress induced by DQ serves as the primary driver of nerve cell death. As long as NADPH and O_2_ are present, the cyclic redox reaction initiated by DQ continues indefinitely. The depletion of NADPH disrupts glutathione circulation and interferes with other intracellular processes, including energy production and active transporters, thus exacerbating neurotoxicity. Additionally, intracellular protective mechanisms such as glutathione, SOD, and CAT are depleted. Antioxidant compounds may offer relief from DQ’s toxic effects on nerve cells. Reduced glutathione (GSH), the primary intracellular reducing agent, has been reported to alleviate DQ-induced oxidative stress [66]. N-acetylcysteine (NAC), with its active sulfhydryl group (-SH), scavenges oxygen free radicals and serves as a glutathione synthesis precursor, effectively replenishing intracellular reduced glutathione levels [67]. However, strict infusion rate control is essential when administering NAC treatment to prevent allergic or anaphylactoid reactions.

Moreover, studies have shown that pretreatment with N-nitro-L-arginine methyl ester arginine (L-NAME), a nonselective inhibitor of nitrate synthase, can reduce the neurotoxicity of DQ poisoning in rats [68]. Resveratrol (RSV) has demonstrated protective effects on the gastrointestinal tract and nerves by mitigating DQ-induced disruption of antioxidant enzymes, reducing ROS production, alleviating mitochondrial depolarization, improving mitochondrial morphology, and inducing mitochondrial phagocytosis [69–73]. Additionally, standardized extracts of Bacopa monnieri have been reported to protect nerve cells from DQ-induced damage by modulating PD-altered cellular redox pathways [54].

Despite our best efforts, current treatments do not guarantee the survival of all patients with DQ poisoning. Therefore, there is an urgent need for an antidote or an effective treatment for DQ poisoning. Potential avenues include conducting in vitro and in vivo studies to evaluate the effects of inducers of efflux transporters and facilitating the release of DQ residues from cells and their excretion through feces or urine to minimize toxicity. Complex formation with DQ may inhibit its cell entry and serve as a detoxification method. For patients with high-dose poisoning, the possibility of central nervous system injury should be anticipated, and cranial imaging (MR or CT) should be promptly conducted. When necessary, cerebrospinal fluid toxicology analysis and cerebrospinal fluid pressure measurement should be performed to enable the early detection of central nervous system lesions and the implementation of timely treatment plans.

## Conclusion and Perspectives

Neurological damage resulting from DQ poisoning is not only common but also clinically severe, often leading to a poor prognosis [68, 74]. The limited research on DQ poisoning in the past can be attributed to the relatively low number of DQ poisoning cases. However, as the use of DQ becomes more widespread, especially as a substitute for PQ, the incidence of DQ poisoning is expected to rise. Consequently, the number of cases involving neurological symptoms after poisoning is also likely to increase.

Although current treatment methods involve the use of antioxidants and symptomatic supportive care, these approaches offer limited benefits for patients with severe neurological symptoms. The precise mechanism underlying neurological damage caused by DQ poisoning remains unclear. However, it has been observed that mitigating the degeneration of dopaminergic neurons in the substantia nigra through the regulation of mitophagy and the modulation of the inflammatory cascade of microglial activation in response to oxidative stress may hold promise. Reducing oxidative stress-mediated axonal degeneration represents a potential avenue for treating DQ nerve injury.

In the future, further research is imperative to elucidate the mechanisms of DQ poisoning. This knowledge can be the foundation for identifying therapeutic targets within the damage mechanism and developing targeted specific antidotes. These efforts are essential to provide more effective treatment for patients with DQ poisoning, ultimately reducing mortality rates and improving the quality of life for those affected.

## Data Availability

No data was used for the research described in the article.
